# Deletion of endothelial arginase 1 does not improve vasomotor function in diabetic mice

**DOI:** 10.14814/phy2.13717

**Published:** 2018-06-10

**Authors:** Ramesh Chennupati, Merlijn J. Meens, Ben J. Janssen, Paul van Dijk, Theodorus B. M. Hakvoort, Wouter H. Lamers, Jo G. R. De Mey, S. Eleonore Koehler

**Affiliations:** ^1^ Departments of Anatomy & Embryology Maastricht University Maastricht the Netherlands; ^2^ Department of Pharmacology & Toxicology Maastricht University Maastricht the Netherlands; ^3^ Cardiovascular Research Institute Maastricht (CARIM) Maastricht University Maastricht the Netherlands; ^4^ Nutrim ‐ School of Nutrition and Translational Research in Metabolism Maastricht University Maastricht the Netherlands; ^5^ Department of Pathology and Immunology University of Geneva Geneva Switzerland; ^6^ Tytgat Institute for Liver & Intestinal Research Academic Medical Center Amsterdam the Netherlands; ^7^ Department of Cardiovascular and Renal Research Institute of Molecular Medicine University of Southern Denmark Odense Denmark; ^8^ Department of Cardiac Thoracic and Vascular Surgery Odense University Hospital Odense Denmark

**Keywords:** Arginase 1 deficiency, endothelial dysfunction, nitric oxide

## Abstract

Endothelial arginase 1 was ablated to assess whether this prevents hyperglycemia‐induced endothelial dysfunction by improving arginine availability for nitric oxide production. Endothelial *Arg1*‐deficient mice (Arg1‐KO^T^ ^ie2^) were generated by crossing *Arg1* ^*fl/fl*^ (controls) with *Tie2Cre* ^*tg/−*^ mice and analyzed by immunohistochemistry, measurements of hemodynamics, and wire myography. Ablation was confirmed by immunohistochemistry. Mean arterial blood pressure was similar in conscious male control and Arg1‐KO^T^ ^ie2^ mice. Depletion of circulating arginine by intravenous infusion of arginase 1 or inhibition of nitric oxide synthase activity with L‐N^G^‐nitro‐arginine methyl ester increased mean arterial pressure similarly in control (9 ± 2 and 34 ± 2 mmHg, respectively) and Arg1‐KO^T^ ^ie2^ mice (11 ± 3 and 38 ± 4 mmHg, respectively). Vasomotor responses were studied in isolated saphenous arteries of 12‐ and 34‐week‐old Arg1‐KO^T^ ^ie2^ and control animals by wire myography. Diabetes was induced in 10‐week‐old control and Arg1‐KO^T^ ^ie2^ mice with streptozotocin, and vasomotor responses were studied 10 weeks later. Optimal arterial diameter, contractile responses to phenylephrine, and relaxing responses to acetylcholine and sodium nitroprusside were similar in normoglycemic control and Arg1‐KO^T^ ^ie2^ mice. The relaxing response to acetylcholine was dependent on the availability of extracellular l‐arginine. In the diabetic mice, arterial relaxation responses to endothelium‐dependent hyperpolarization and to exogenous nitric oxide were impaired. The data show that endothelial ablation of arginase 1 in mice does not markedly modify smooth muscle and endothelial functions of a resistance artery under normo‐ and hyperglycemic conditions.

## Introduction

Vascular complications are the primary cause of morbidity and mortality in diabetics. Endothelial dysfunction is considered a critical factor in the development of these complications (Luscher et al. 2003; Sena et al. 2013). One aspect of endothelial dysfunction is a decrease in the bioavailability of endothelium‐derived nitric oxide (EDNO). This decrease may be caused by lowered endothelial nitric oxide synthase (NOS3) activity, increased levels of endogenous NOS inhibitors, limited availability of the NOS3 substrate arginine (Luscher et al. 2003; Cooke et al. 1991; Wu and Meininger 2000), or increased scavenging of nitric oxide (NO) by reactive oxygen species (ROS) (Gryglewski et al. 1986). Under healthy, physiological conditions, sufficient L‐arginine is available for NO production in the endothelium. However, this may be altered in pathological conditions, such as diabetes, where arginine availability for NO synthesis may be becoming limited. We previously showed that under diabetic conditions arginine resynthesis from citrulline is necessary to maintain arginine supply for NO‐mediated arterial relaxation (Chennupati et al. 2014).

Arginine‐resynthesizing enzymes (i.e., the enzymes of the citrulline‐NO cycle) and arginine‐catabolizing enzymes (arginases, ARG) tightly regulate intracellular arginine concentrations (Li et al. 2001). The two arginase isoforms, ARG1 and ARG2, have similar kinetic properties, but are encoded by separate genes. Both ARG1 and ARG2 proteins were demonstrated in endothelium (for a review, see Pernow and Jung (2013)) and implicated in endothelial dysfunction. Increased *Arg1* expression is associated with endothelial dysfunction in diabetic patients (Bagi et al. 2013; Beleznai et al. 2011) and mice (Romero et al. 2012; Toque et al. 2013a).

To evaluate roles of arginases, three constitutive knockout mouse models were previously developed: *Arg1* ^*−/−*^ *(*Iyer et al. 2002), *Arg2* ^*−/−*^ (Shi et al. 2001), and double knockout (KO) mice (Deignan et al. 2006). *Arg* ^*−/−*^ and double KO mice develop severe hyperammonemia and die within 10–14 days after birth (Iyer et al. 2002). Intriguingly, heterozygous, constitutive partial ablation of ARG1 (*Arg1* ^*+/−*^), complete deletion of ARG2 (*Arg2* ^*−/−*^), and the combination of these two genotypes (*Arg1* ^*+/−*^ */Arg2* ^*−/−*^) partially rescue diabetes‐induced, endothelial dysfunction in mouse aorta and corpus cavernosum (Romero et al. 2012; Toque et al. 2011). Because *Arg1* ^*−/−*^ mice do not survive beyond 2 weeks after birth, the role of ARG1 in vascular dysfunction has not been investigated. We hypothesized that limitation of the capacity of the endothelium to degrade arginine via ARG1 improves arterial endothelium‐dependent relaxation, especially when NO‐mediated relaxations are compromised (as in diabetic mice). This question is pertinent because ARG1 and NOS3 compete for the same substrate. We tested this hypothesis in saphenous arteries of normoglycemic and diabetic mice that did or did not express ARG1 in their endothelium, because these muscular resistance arteries are sensitive to impairment of the NO‐citrulline cycle (Chennupati et al. 2014).

## Materials and Methods

### Animals

All procedures were approved by the Committee for Animal Care and Use of Maastricht University (DEC 2008‐182 and 2012‐027) and performed in accordance with their guidelines. Endothelial ablation of ARG1 was achieved by crossing *Arg1* ^*fl/fl*^(Cloots et al. 2013) and *Tie2Cre* ^*tg/−*^(Kisanuki et al. 2001) mice. Mice that lack ARG1 in their endothelium (*Arg1* ^*fl/fl*^ */Tie2Cre* ^*tg/−*^) are named Arg1‐KO^Tie2^ hereafter. We have previously shown that mice carrying the fully functional floxed alleles of the ARG1 gene (*Arg1* ^*fl/fl*^) are indistinguishable from their wild‐type littermates (Cloots et al. 2013). Therefore, *Arg1* ^*fl/fl*^ */Tie2Cre* ^*−/−*^ littermates were used as control animals; 12‐ and 34‐week‐old male and female mice were used. Mice were housed in standard cages (constant room temperature and humidity, 12 h light/dark cycles) and had free access to standard pelleted chow and tap water.

Diabetes was induced at the age of 10 weeks by intraperitoneal (IP) injections of streptozotocin (STZ; 50 mg·kg^−1^) on 5 consecutive days (American diabetes complications consortium AMDCC protocols; https://www.diacomp.org). Blood glucose was measured following overnight fasting at 1, 4, and 10 weeks following STZ injections (Table S5), and male mice with stable blood glucose concentration of ≥20 mmol·L^−1^ were used for the experiments (mean ± SEM: controls 22.8 ± 0.8 mmol·L^−1^, *n* = 10 (Chennupati et al. 2014); Arg1‐KO^Tie2^ 23.1 ± 0.6 mmol·L^−1^, *n* = 5). Female mice were not included in the experiments because their fasting blood glucose concentrations only transiently increased to ~60% of the values measured in males at 1 and 4 weeks after STZ treatment and returned to pretreatment values between 4 and 10 weeks (mean ± SEM at 10 weeks: controls 7.8 ± 1.0 mmol·L^−1^, *n* = 8; Arg1‐KO^Tie2^ 7.6 ± 0.9 mmol·L^−1^, *n* = 4; Table S5; cf Chennupati et al. 2014). This finding suggests that the *β* cells of female mice are more resistant to STZ and regenerate between 4 and 10 weeks after the treatment.

### Solutions and drugs

Krebs‐Ringer bicarbonate‐buffered salt solution (KRB) contained (in mmol·L^−1^): 118.5 NaCl, 4.7 KCl, 2.5 CaCl_2_, 1.2 MgSO_4_, 1.2 KH_2_PO_4_, 25.0 NaHCO_3_, and 5.5 glucose. The KRB solution was continuously aerated with 95% O_2_/5% CO_2_ and maintained at 37°C. Indomethacin (INDO; Sigma Aldrich, Zwijndrecht, NL) was dissolved in ethanol. Acetylcholine (ACh), noradrenaline (NA), phenylephrine (PHE), N^***ω***^‐nitro‐arginine methyl ester (L‐NAME), and sodium nitroprusside (SNP; all Sigma Aldrich) were dissolved in KRB solution. High K^+^‐KRB solution was prepared by replacing 40 mmol·L^−1^ NaCl with KCl. Buffers containing intermediate K^+^ concentrations were prepared by mixing KRB and K^+^‐KRB. In the l‐arginine‐depletion experiment (Fig. 4), KRB was replaced by l‐arginine‐free Iscove's modified Dulbecco's medium.

### Plasma amino acid analysis

To determine plasma amino acids, blood was drawn from the inferior vena cava using a heparinized syringe, centrifuged, deproteinized by adding 50 *μ*L plasma to tubes containing 4 mg sulfosalicylic acid, and stored at −80°C. Amino acid concentrations were determined by HPLC (van Eijk et al. 1993).

### Immunohistochemistry

Saphenous arteries were fixed in formaldehyde (4%) or cold acetone/methanol/water (2:2:1; 4°C) overnight, dehydrated, and embedded in paraffin. Sections (4 *μ*m) were incubated overnight at 4°C with rabbit antibodies directed against ARG1 (1:10,000 in 10% normal goat serum NGS; AMC, Amsterdam; de Jonge et al. 2002). Antibody binding was visualized with alkaline phosphatase (AP)‐coupled goat anti‐rabbit IgG antibodies (diluted 1:200 in Teng‐T/10% NGS: 100 mmol·L^−1^ Tris‐HCl, pH 8.0, 50 mmol·L^−1^ EDTA, 1.5 mmol·L^−1^ NaCl, 2.5% (w/v) gelatin, 0.5% (v/v) Tween^®^ 20, 10% (v/v) NGS) and incubation with AP substrate (NBT/BCIP) for 15–90 min at room temperature. Negative controls were incubated with secondary antibody only. Background staining was equalized by globally adapting the brightness of colors with Adobe Photoshop, with panels C and G serving as reference. The contrast was modified in the H&E‐stained panels E and H only.

### Determination and manipulation of hemodynamic parameters

We determined hemodynamic parameters in conscious, unrestrained control, and Arg1‐KO^Tie2^ mice. Heparinized indwelling polyethylene catheters were introduced into the femoral artery and jugular vein under isoflurane anesthesia 2 days before the experiments (Janssen et al. 2000). Analgesia was obtained by perioperative subcutaneous injections of buprenorphine (0.03 mg·kg^−1^). On the day of the experiment, the arterial line was connected to a pressure transducer (Micro Switch 150 PC) and the signal was sampled at 2.5 kHz. Mean arterial pressure (MAP) and heart rate (HR) were derived from this signal using the IDEEQ data acquisition system (Instrument Services, Maastricht University, NL). The venous line was extended outside the cage and filled with a 0.9% NaCl solution. Hemodynamic parameters were allowed to stabilize before pharmacological interventions. The following compounds were applied via an intravenous catheter: 200 U purified bovine ARG1 (Cell Sciences, Canton, MA, USA) dissolved in 125 *μ*L HEPES buffer (pH 7.4) or L‐NAME (10 mg·kg^−1^) dissolved in 0.9% saline. After the experiments, animals were euthanized with 250 mg·kg^−1^ pentobarbital administered through the catheter. Cannulation of femoral arteries in diabetic mice is associated with a full occlusion of the main feeding artery of the leg. In those mice, wound healing and revascularization are so much impaired that the leg becomes necrotic. For this reason, diabetic mice could not be subjected to the same protocol as the nondiabetic mice.

### Organ chamber experiments

Animals were euthanized by CO_2_/O_2_ inhalation. Saphenous arteries were dissected free from perivascular adipose tissue and mounted in a wire myograph (Danish Myotechnology, Aarhus, DK). Arterial segments (2 mm) were distended to the diameter at which maximal contractile responses to 10 *μ*mol·L^−1^ NA were obtained (Chennupati et al. 2013; Hilgers et al. 2010). Optimal diameters (D_opt_) and maximal contractile responses to NA are summarized in Table S2.

### Contributions of NO, endothelium‐dependent hyperpolarization (EDH) and cyclooxygenase products to endothelium‐dependent relaxation

Initially, a concentration‐response curve (CRC) for PHE (0.01–10 *μ*mol·L^−1^) was recorded. Following the contraction induced by 10 *μ*mol·L^−1^ PHE, an ACh CRC (0.01–10 *μ*mol·L^−1^) was generated. Next, arteries were contracted using K^+^ (40 mmol·L^−1^), and again an ACh CRC (0.01–10 *μ*mol·L^−1^) was recorded. These experiments were repeated in the presence of the cyclooxygenase inhibitor INDO (10 *μ*mol·L^−1^) and in the presence of both INDO and the NOS inhibitor L‐NAME (100 *μ*mol·L^−1^) to assess the contribution of NO to arterial relaxation (Morikawa et al. 2005; Matoba et al. 2000).

### Sensitivity of vascular smooth muscle to NO

During contraction of the saphenous arteries with PHE (10 *μ*mol·L^−1^) in the presence of INDO (10 *μ*mol·L^−1^) and L‐NAME (100 *μ*mol·L^−1^), the relaxing effects of the NO‐donor SNP (0.01–10 *μ*mol·L^−1^) were recorded.

### Statistical analysis

All CRCs for contractile stimuli are expressed as percentage of the maximal response to 10 *μ*mol·L^−1^ NA prior to the administration of pharmacological inhibitors. Relaxing responses were expressed as percentage reduction of the level of contraction. Individual CRCs were fitted to a nonlinear sigmoid regression curve (Graphpad Prism 5.0). Sensitivity, maximal effect (Maximal response), and amino acid concentrations are shown as means ± SEM. Two‐way ANOVA followed by a Bonferroni post hoc test was used to compare multiple groups. Group statistics for hemodynamic parameters were analyzed by an independent samples test to determine differences between genotypes, followed by paired *t*‐tests to assess the effects of treatments.

## Results

### Ablation of ARG1 protein from endothelial cells

**Figure 1:**
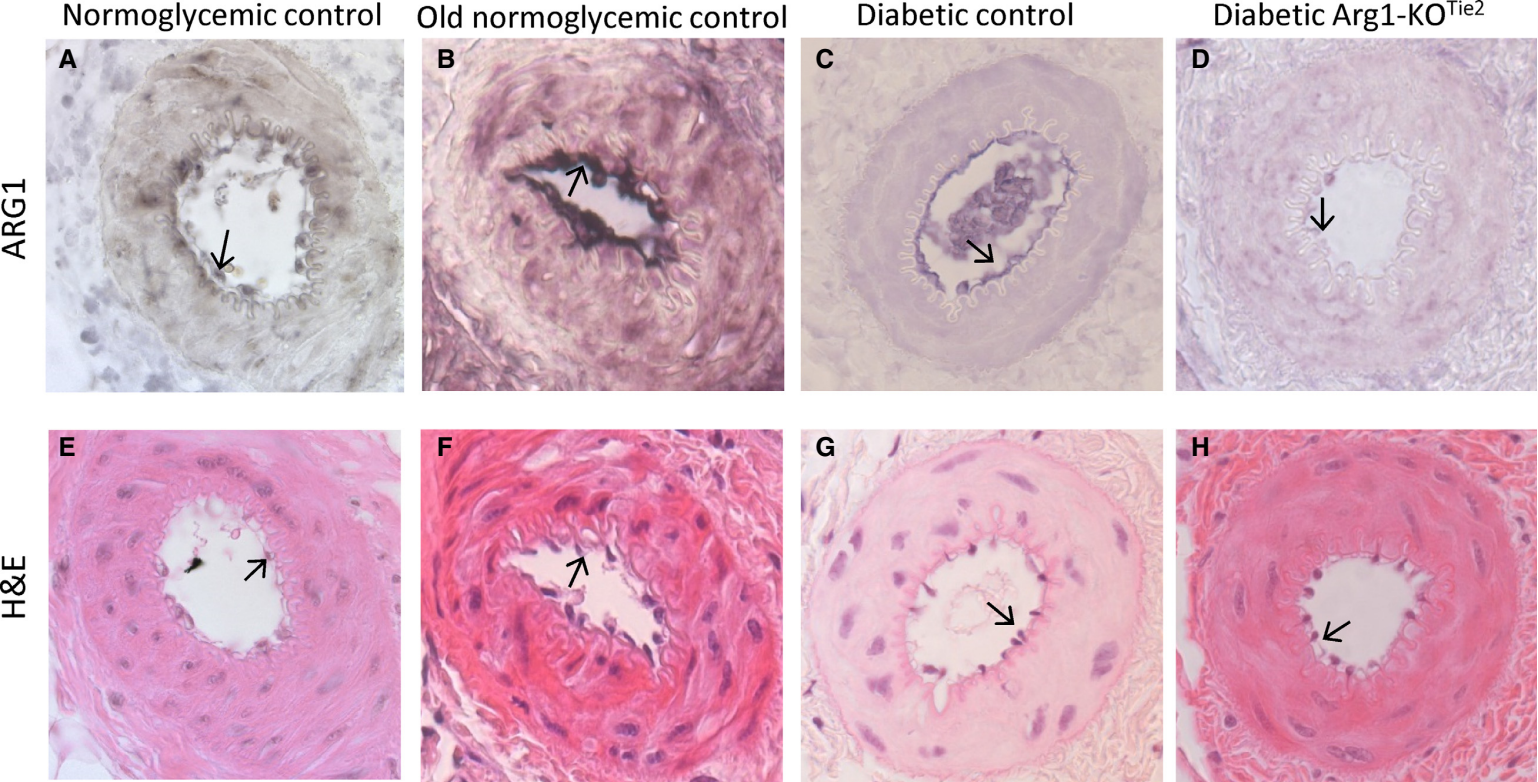
Expression of ARG1 protein in saphenous arteries of male mice. Panel (A): control, panel (B): 8‐month‐old normoglycemic control, panel (C): diabetic control (DM), panel (D): diabetic Arg1‐KO^T^ ^ie2^ mice. Panels (E, F, G) and (H) show the complementary H&E staining of a serial section of the respective arteries. Arrows indicate endothelial cells. All tissues were fixed with acetone/methanol/water (see Materials and Methods).

Endothelial ablation of ARG1 was achieved by crossing *Arg1* ^*fl/fl*^ and *Tie2Cre* ^*tg/−*^ mice. Mice that lack ARG1 in their endothelium (*Arg1* ^*fl/fl*^ */Tie2Cre* ^*tg/−*^) are named Arg1‐KO^Tie2^ hereafter. We have previously shown that mice carrying the fully functional floxed alleles of the ARG1 gene (*Arg1* ^*fl/fl*^) are indistinguishable from their wild‐type littermates (Cloots et al. 2013). Therefore, *Arg1* ^*fl/fl*^ */Tie2Cre* ^*−/−*^ littermates were used as control animals. To verify endothelial deletion of ARG1 protein, saphenous artery sections were stained for ARG1. The sensitivity of immunohistochemical staining for ARG1 in endothelial cells was much improved by fixation of the tissues in acetone/methanol/water (2:2:1) instead of formaldehyde. In young adult control mice, endothelial ARG1 was weakly expressed (Fig. 1A and E). In line with earlier observations (Romero et al. 2012; Toque et al. 2013a; White et al. 2006; Pernow et al. 2015), we observed stronger expression of ARG1 in arteries of 34‐week‐old control (Fig. 1B and F) and 22‐week‐old diabetic mice (Fig. 1C and G), but not in diabetic Arg1‐KO^Tie2^ mice (Fig. 1D and H). Specificity of the antibody‐staining procedure was demonstrated by zonated ARG1 expression in liver sections (Fig. S1) (Dingemanse et al. 1996).

### Plasma amino acid concentrations in control and Arg1‐KO^Tie2^ mice

Especially in diabetes, upregulation of arginase expression can limit the availability of arginine for NOS in aging vessels (Romero et al. 2012; Toque et al. 2013a; Pernow et al. 2015; Santhanam et al. 2008). Thus, absence of endothelial ARG1 might improve endothelial function by increasing arginine availability for NOS3 in diabetic mice. We only studied male diabetic mice, because the AMDCC protocol induced only transient hyperglycemia in female C57BL/6 mice (see M&M section). Plasma arginine concentrations were similar in control and Arg1‐KO^Tie2^ mice (Tables 1 and S1). Despite increased ARG1 expression in the endothelium of diabetic control mice, their plasma arginine concentration was comparable to that of normoglycemic control mice. In diabetic Arg1‐KO^Tie2^ mice, however, the plasma arginine concentration was increased (~30%; *P *=* *0.030). The concentration of the arginine precursor citrulline was increased in diabetic mice compared with normoglycemic mice, while that of tryptophan was significantly decreased in diabetic Arg1‐KO^Tie2^ mice, and a trend (*P *=* *0.054) toward a decrease was observed in diabetic control mice (Tables 1 and S1).

**Table 1:** Effect of *Arg1* ablation on plasma amino acid concentrations in 12‐week‐old healthy male and 22‐week‐old male diabetic mice

Plasma amino acids [*μ*mol·L]	Control	Arg1‐KO^Tie2^	Control STZ‐treated	Arg1‐KO^Tie2^ STZ‐treated
Alanine	484 ± 25	390 ± 72	500 ± 51	575 ± 67
Arginine	103 ± 13	126 ± 26	121 ± 9	156 ± 8^1^
Asparagine	46 ± 15	33 ± 11	39 ± 5	61 ± 13
Ornithine	91 ± 4	79 ± 8	100 ± 9	116 ± 15
Citrulline	52 ± 5	46 ± 4	78 ± 5^2^	85 ± 7^2^
Glutamic acid	88 ± 9	80 ± 15	97 ± 10	110 ± 14
Glutamine	518 ± 80	518 ± 102	537 ± 29	577 ± 55
Glycine	276 ± 16	260 ± 35	236 ± 13	275 ± 26
Histidine	71 ± 5	61 ± 5	56 ± 5	59 ± 4
Isoleucine	97 ± 10	87 ± 8	170 ± 21	192 ± 42
Leucine	159 ± 11	154 ± 14	279 ± 36	313 ± 68
Lysine	256 ± 20	256 ± 32	336 ± 67	485 ± 78
Methionine	43 ± 1	40 ± 4	37 ± 4	48 ± 5
Phenylalanine	43 ± 10	59 ± 5	59 ± 3	72 ± 7
Taurine	72 ± 11	73 ± 12	80 ± 6	131 ± 15^2^^**,**^ 1
Serine	132 ± 16	115 ± 17	129 ± 11	149 ± 21
Threonine	132 ± 17	116 ± 15	140 ± 13	169 ± 21
Tryptophan	259 ± 21	255 ± 33	169 ± 19	159 ± 16^2^
Tyrosine	332 ± 31	404 ± 43	312 ± 38	306 ± 27
Valine	259 ± 15	220 ± 13	387 ± 49	432 ± 90
ΣAA	3578 ± 256	3449 ± 407	3863 ± 215	4851 ± 554
Arginine‐availability index	0.33 ± 0.03	0.32 ± 0.02	0.36 ± 0.05	0.26 ± 0.03

All values are shown as means ± SEM.

^1^ *P *<* *0.05 compared with the corresponding control group (knockout vs. wild type).

^2^ *P *<* *0.05 compared with the corresponding healthy group (diabetes vs. healthy). Control: *n* = 3; Arg1‐KO^Tie2^: *n* = 4; STZ‐control: *n* = 14; STZ‐Arg1‐KO^Tie2^: *n* = 6. Arginine‐availability index: [Arg]/([Orn] + [Lys]). For *P*‐values of the comparisons, see Table S1.

### Hemodynamic parameters in male control and Arg1‐KO^Tie2^ mice

**Figure 2:**
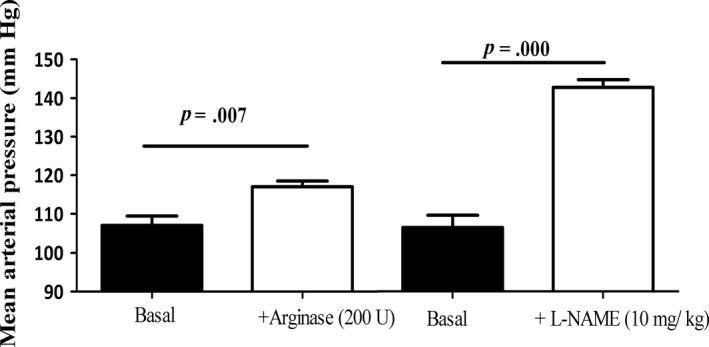
Effects of endothelial *Arg1* ablation on mean arterial blood pressure (MAP) in 34‐week‐old conscious male mice. Blood pressure was measured in the same mice 2 (Basal, +Arginase) and 3 days (Basal 2, +L‐NAME) after implantation of a femoral artery catheter connected to a pressure transducer. Since MAP in control and Arg1‐KO^T^ ^ie2^ mice did not differ either under basal or treatment conditions, data from both groups were pooled. Left side: MAP under basal conditions and after a bolus injection of 200U bovine ARG1 via a jugular vein catheter. Right side: MAP under basal conditions one day later (Basal 2) and after intravenous L‐NAME (10 mg·kg^−1^) injection (right). Values are means ± SEM. Nine mice (five control + four Arg1‐KO^T^ ^ie2^) received the arginase infusion; due to loss of catheter patency in one control mouse, eight mice received the L‐NAME infusion. Differences between treatments were analyzed with a paired *t*‐test.

To assess the effects of endothelial *Arg1* ablation on hemodynamics, mean arterial pressure (MAP) and heart rate (HR) were recorded in conscious male mice. Basal MAP did not differ between conscious unrestrained control and Arg1‐KO^Tie2^ mice (*P *=* *0.777). MAP after arginase infusion did not differ between control and Arg1‐KO^Tie2^ mice (*P *=* *0.257), demonstrating that endothelial *Arg1* ablation does not influence the hemodynamic effects of circulating arginine. Accordingly, data from both groups were pooled. The effects of circulating arginine on blood pressure maintenance were assessed by a bolus injection of 200U ARG1, which rapidly decreases the concentration of circulating arginine to ~13% of the original plasma concentration during at least 20 min (Chennupati et al. 2014; Wijnands et al. 2015). This intervention resulted in a small but significant rise of MAP to 108% of control values (9 ± 2.5 mmHg increase, *P *=* *0.007; Fig. 2). Again, control and Arg1‐KO^Tie2^ mice did not differ significantly (*P *=* *0.115). To compare the effects of arginine depletion to those of NOS inhibition, the same animals received a bolus injection of the NOS inhibitor L‐NAME (10 mg·kg^−1^) 24 h later. This treatment resulted in a 36 ± 2 mmHg increase in MAP (*P *<* *0.000), that is, in a fourfold higher increase than that caused by plasma arginine depletion. No significant differences were observed between knockout and wild‐type animals.

Basal HR did not differ between control (651 ± 21 beats/min, *n* = 6) and Arg1‐KO^Tie2^ mice (713 ± 42 beats/min, *n* = 6, *P *=* *0.186, Table 2). The decrease in circulating arginine concentration upon injection of 200U ARG1 (previous paragraph) had no significant effect on HR and again, and no difference between genotypes was observed. Accordingly, data of control and Arg1‐KO^Tie2^ animals were pooled (Table 2). HR decreased to a similar extent in L‐NAME‐treated control and Arg1‐KO^Tie2^ mice (−189 ± 46/min, *n* = 7, *P *=* *0.006).

**Table 2:** Effects of genotype, arginase 1, and L‐NAME treatment on heart rate in conscious male mice

Comparison of genotypes				Effect of treatment		
	Control [beats. min^−1^]	ARG1‐KO^Tie2^ [beats. min^−1^]	*P*		ΔHR [bp. min^−1^]	*P*
Basal HR	651 ± 21	713 ± 42	0.184			
Before ARG1	657 ± 37	733 ± 45	0.230	ARG1	39 ± 33	0.276
After ARG1	627 ± 35	693 ± 49	0.326			
Before L‐Name	661 ± 30	768 ± 48	0.109	L‐NAME	189 ± 46	0.006
After L‐NAME	525 ± 15	531 ± 66	0.914			

Heart rate was determined as described in Materials and Methods. Values are given as means ± SEM. Differences between genotypes were assessed with an independent *t*‐test (*n* per group 3–5 animals). Since control and Arg1‐KO^Tie2^ mice did not differ significantly under any of the conditions tested, all animals were pooled to assess the effect of the treatments with a paired *t*‐test (*n* for ARG1 = 9, L‐NAME = 7).

### Reactivity of isolated saphenous arteries

Endothelial ARG1 deficiency might entail compensatory mechanisms such as increased arterial contractility or decreased vasodilator responses to maintain blood pressure. We, therefore, studied the effects of endothelial *Arg1* ablation on vasomotor responses in more detail in saphenous arteries isolated from male and female mice of 12‐ and 34‐week‐old control and Arg1‐KO^Tie2^ mice, and 22‐week‐old male STZ‐treated mice of both genotypes.

#### Contractile responses of control and Arg1‐KO^Tie2^ arteries

The maximal contractile response to 10 *μ*mol·L^−1^ noradrenaline (NA) was comparable in 12‐ and 34‐week‐old male and female control and Arg1‐KO^Tie2^ mice (Tables S2 (male); S5 (female mice). The sensitivity and maximal contractile response to phenylephrine (PHE) (0.01–10 *μ*mol·L^−1^) or K^+^ (40 mmol·L^−1^) in the absence and presence of L‐NAME (100 *μ*mol·L^‐1^) or indomethacin (cyclooxygenase inhibitor, INDO; 10 *μ*mol·L^‐1^) and a combination of the former did also not differ significantly between groups (Tables S2, S5). Contractile responses did not differ significantly between saphenous arteries from male diabetic Arg1‐KO^Tie2^ and control mice (Table S2), showing that a decreased capacity for endothelial arginine degradation does not improve contractile responses of vessels in diabetic mice.

#### Acetylcholine (ACh)‐induced relaxations during PHE‐induced contractions

**Figure 3:**
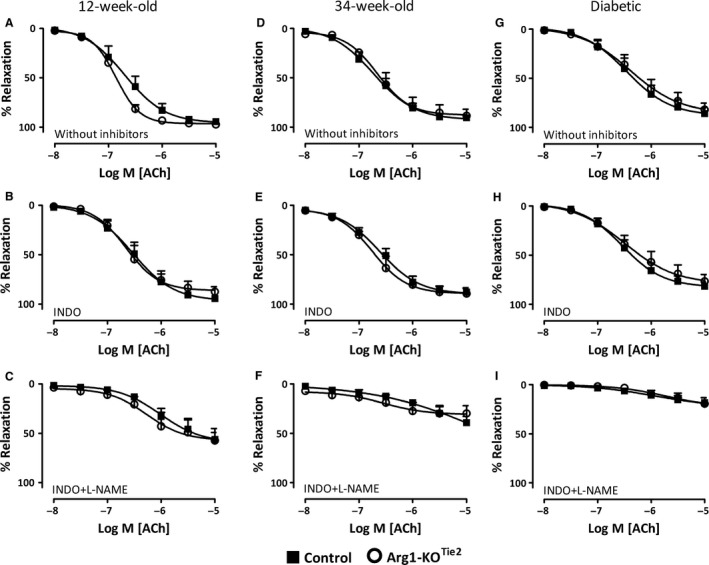
Effects of endothelial *Arg1* ablation on relaxation responses to ACh (0.01–10 *μ*mol·L^−1^) during PHE‐ (10 *μ*mol·L^−1^) induced contractions in saphenous arteries isolated from 12‐ (panels A–C) or 34‐ (panels D–F) week‐old normoglycemic and 22‐week‐old hyperglycemic (panels G–I) male mice. Black squares: control mice; white circles: Arg1‐KO^T^ ^ie2^ mice. Panels (A, D, G) in the absence of pharmacological inhibitors. Panels (B, E, H) in the presence of indomethacin (INDO, 10 *μ*mol.L^−1^). Panels (C, F, I) in the presence of INDO (10 *μ*mol·L^−1^) and L‐NAME (100 *μ*mol·L^−1^). Values are shown as means ± SEM (*n* = 5–7; for the number of animals per individual experiment, see Table S2).

ACh‐induced relaxations were studied during PHE‐induced contractions. In the absence of pharmacological inhibitors and in the presence of INDO, the maximal response and sensitivity to ACh were similar in control and Arg1‐KO^Tie2^ mice of both sexes and both age groups (Fig. 3A and D male mice and S2 female mice, Tables 3, S3, S4, S5). INDO had no statistically significant effects on ACh‐relaxation irrespective of genotype or age (Fig. 3A, B, D and E). The combination of INDO and L‐LAME reduced ACh‐induced relaxations to a similar degree in all groups of nondiabetic arteries (Fig. 3B, C, E and F). Thus, in control arteries, the presence or absence of endothelial ARG1 does not affect endothelium‐dependent relaxation. As shown earlier in mesenteric arteries (Morikawa et al. 2005), diabetes reduced the relaxation responses in saphenous arteries (Fig. 3G, H and I). However, the maximal response and sensitivity of ACh‐induced relaxations did not differ between diabetic control and diabetic Arg1‐KO^Tie2^ mice in any of conditions tested (Fig. 3G, H and I).

**Table 3:** Effect of endothelial ablation of *Arg1* on relaxation responses

	Control			Arg1‐KO^Tie2^		
	Sensitivity	Maximal response%	*n*	Sensitivity	Maximal response%	*n*
12‐week‐old mice						
Without inhibitors	6.7 ± 0.1	94 ± 2	7	6.7 ± 0.1	97 ± 3	4
INDO	6.5 ± 0.2	94 ± 1	6	6.6 ± 0.1	88 ± 4	5
INDO + L‐NAME	6.0 ± 0.1	56 ± 7	7	6.2 ± 0.2	57 ± 12	5
Relaxation to EDNO	6.1 ± 0.1	60 ± 4	6	5.9 ± 0.1	56 ± 6	4
Relaxation to SNP	7.6 ± 0.1	97 ± 1	5	7.7 ± 0.1	97 ± 1	5
34‐week‐old mice						
Without inhibitors	6.7 ± 0.1	90 ± 3	7	6.6 ± 0.1	88 ± 6	4
INDO	6.6 ± 0.1	88 ± 3	8	6.7 ± 0.3	89 ± 6	4
INDO + L‐NAME	NA	39 ± 6	7	NA	29 ± 8	4
Relaxation to EDNO	6.1 ± 0.2	54 ± 7	6	5.8 ± 0.2	53 ± 6	4
Relaxation to SNP	7.5 ± 0.1	97 ± 1	7	7.6 ± 0.3	98 ± 1	5
22‐week‐old diabetic mice						
Without inhibitors	6.5 ± 0.1	86 ± 6	7	6.4 ± 0.2	82 ± 8	5
INDO	6.5 ± 0.1	81 ± 4	8	6.5 ± 0.2	76 ± 7	5
INDO + L‐NAME	NA	18 ± 5	7	NA	19 ± 6	5
Relaxation to EDNO	6.2 ± 0.1	47 ± 3	6	6.0 ± 0.2	41 ± 6	5
Relaxation to SNP	7.0 ± 0.1^2^	98 ± 1	7	6.8 ± 0.1^1^,^2^	96 ± 1	5

Relaxation responses to acetylcholine (ACh; 0.01–10 *μ*mol·L^−1^) in PHE‐ (10 *μ*mol·L^−1^) or K^+^ (40 mmol·L^−1^) contracted vessels were determined in the presence or absence of indomethacin (INDO, 10 *μ*mol·L^−1^) and L‐NAME (100 *μ*mol·L^−1^). Maximal relaxation (Maximal response) is expressed as % reduction of the maximal contractile response to PHE (10 *μ*mol·L^−1^), except for endothelium‐derived nitric oxide (EDNO) responses (% reduction of maximal contractile response to 40 mmol·L^−1 ^K^+^). In the presence of NOS inhibitor L‐NAME (100 *μ*mol·L^−1^) and INDO (10 *μ*mol·L^−1^), the maximal relaxation to an NO‐donor is determined by exposing the vessels to SNP (0.01–10 *μ*mol·L^−1^).

^1^ *P *<* *0.05 compared with the corresponding control group (knockout vs. wild type).

^2^ *P* < 0.05 compared with the corresponding control group (diabetes vs. control). All values are shown as mean ± SEM.

**Figure 4:**
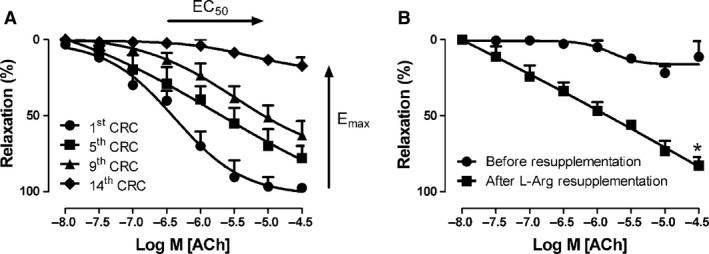
The effect of 14 consecutive contraction‐relaxation cycles (CRC) on ACh‐induced relaxation during contractions induced by PHE (10 *μ*mol·L^−1^). Two‐mm arterial segments were kept in L‐Arg‐free culture medium during the experiment. The EC_50_ increased, whereas the Maximal response decreased with increasing number of CRCs (panel A). In a separate experiment (panel B), the arterial segments were subjected to repeated CRCs in L‐Arg‐free culture medium until relaxation had decreased to ~25%. L‐Arg (1 mmol·L^−1^) was then added. ACh‐induced relaxations were tested after 60 min. CRC: contraction‐relaxation cycle.

In view of the above‐described lack of an effect of genotype on ACh‐induced arterial relaxations, we studied whether the extracellular l‐arginine concentration can be limiting for these relaxations in control mice. We, therefore, subjected isolated arteries in L‐Arg‐free Iscove's modified Dulbecco's medium to 14 consecutive contraction‐relaxation cycles at 20‐min intervals. Figure 4A shows that the sensitivity and maximal response to ACh progressively decreased. To demonstrate that this was due to l‐arginine depletion, we repeated the experiment and added l‐arginine to the culture medium after the last contraction‐relaxation cycle (Fig. 4B). Partial restoration of the relaxing response to ACh demonstrates that the decline of the relaxing response indeed resulted from L‐Arg depletion.

### Endothelium‐derived NO

Endothelium‐derived NO (EDNO) contributes to endothelium‐dependent relaxation of muscular resistance arteries (Chennupati et al. 2013). To test for differences in EDNO release, we inhibited endothelium‐dependent hyperpolarization (EDH) by depolarizing the vessels with high potassium buffer (40 mmol·L^−1^ K^+^) and by inhibiting cyclooxygenases using INDO (Chennupati et al. 2013). Under these conditions, relaxing responses to ACh can entirely be attributed to NO (Chennupati et al. 2013). These responses were comparable in all groups of mice (Fig. S2; Tables 3 and S3).

### Relaxing responses to SNP

**Figure 5:**
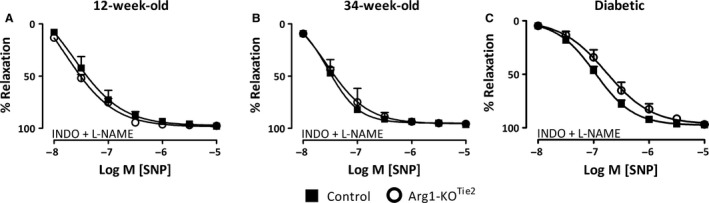
The effect of endothelial *Arg1* ablation on relaxation responses to sodium nitroprusside (SNP, 0.01–10 *μ*mol·L^−1^) during PHE‐ (10 *μ*mol·L^−1^) induced contraction in saphenous arteries of 12‐ (panel A), 34‐ (panel B) week‐old normoglycemic and 22‐week‐old diabetic (panel C) male mice. Black squares: control mice; white circles: Arg1‐KO^T^ ^ie2^. All experiments were performed in the presence of INDO (10 *μ*mol·L^−1^) and L‐NAME (100 *μ*mol·L^−1^). Values are shown as means ± SEM (*n* = 5–7; for the number of animals per individual experiment, see Table S2).

To study that the genetic modification did not affect responses of vascular smooth muscle cells to exogenous NO relaxations in response to the NO‐donor sodium nitroprusside (SNP; 0.01–10 *μ*mol·L^−1^) were studied during PHE‐induced contractions in the presence of INDO and L‐NAME. Sensitivity and maximal response to SNP were comparable between control and Arg1‐KO^Tie2^ mice of both age groups and sexes (Fig. 5, Tables 3, S3 and S4). However, hyperglycemia resulted in a reduced sensitivity (sensitivity: 7.0 ± 0.1) to SNP compared with 12‐ and 34‐week‐old normoglycemic control mice (sensitivity: 7.6 ± 0.1 and 7.5 ± 0.1, respectively; Fig. 5, Tables 2 and S3), as was described earlier (Csanyi et al. 2007). Ablation of endothelial arginase 1 (Arg1‐KO^Tie2^) further reduced arterial smooth muscle sensitivity to SNP (*P *=* *0.045; Fig. 5C, Tables 3 and S3).

## Discussion

In this study, we tested the hypothesis that endothelial ablation of the *Arg1* gene increases endothelium‐dependent vasodilatation in muscular resistance arteries of control and diabetic mice. The major findings of this study are that (1) diabetic Arg1‐KO^Tie2^ mice have a higher arginine concentration in plasma, (2) hemodynamics are not affected by endothelial ARG1 ablation, (3) vasomotor function is not affected by endothelial ARG1 deficiency in normoglycemic mice, and (4) diabetes‐induced alterations in arterial smooth muscle reactivity and endothelium‐dependent relaxations are not prevented by endothelial ARG1 ablation.

In this study, we used the *Tie2‐Cre* transgene (Kisanuki et al. 2001) to eliminate floxed *Arg1* from the endothelium. This transgene has an excellent reputation in eliminating floxed DNA sequences (Kisanuki et al. 2001, 2010; Hamada et al. 2005; Koni et al. 2001; Schlaeger et al. 2005) and, accordingly, was also effective in eliminating floxed *Ass* from the endothelium of the saphenous arteries in C57BL/6 mice (Chennupati et al. 2014). That study showed that resynthesis of arginine from citrulline was necessary to provide sufficient arginine for NO synthesis in saphenous arteries of diabetic mice (Chennupati et al. 2014). Furthermore, we showed earlier that lipopolysaccharide‐treated Arg1‐KO^Tie2^ mice produced more NO in the endothelium of their carotid arteries and jejunum than control mice (Wijnands et al. 2014). In addition, Arg1‐KO^Tie2^ mice suffering from allergic asthma did not express ARG1 mRNA or protein in their lungs, even though this condition upregulates *Arg1* expression >200‐fold in control mice (Cloots et al. 2013). These data underscore our claim that *Arg1* expression was eliminated from the endothelium.

The *Tie2‐Cre* transgene is best known for its expression in cells of hematopoietic origin, such as endothelial cells and macrophages (Tang et al. 2010). Thus, in our Arg1‐KO^Tie2^ mice, cells derived from the hematopoietic lineage are also devoid of ARG1, and this may have effects on circulating concentrations of citrulline and arginine. Therefore, a contribution of *Arg1* elimination in macrophages and/or erythrocytes to blood pressure responses cannot be ruled out. In fact, in a previous study with these mice, we observed effects of *Arg1* ablation that could be attributed to either macrophages (cytokine production) or endothelium (NOS‐dependent effects on the mucosal perfusion of villi in the jejunum) (Wijnands et al. 2014). However, it is highly unlikely that substantial numbers of erythrocytes and macrophages were present in the walls of the vessels used in the wire‐myograph measurements. Ablation of Arg1 in cells of the myeloid lineage has been shown to increase T_H_2‐mediated inflammatory responses in mouse models of schistosoma infection (Pesce et al. 2009). It is, therefore, possible that potential beneficial effects of endothelial Arg1 ablation are masked by increased vascular inflammation due to Arg1 ablation in cells of the myeloid lineage in those same mice, even though we did not find histological evidence of increased vascular inflammation in the vessel walls and surrounding tissue. In a model of allergic asthma in the same mouse strain, we did not find an effect of Arg1 ablation on pulmonary inflammation as determined by cytokine expression and histology (Cloots et al. 2013). We did, however, not investigate cytokine profiles, NO production or leukocyte adhesion to vessel walls in the current model and can, therefore, not exclude that some vascular inflammation was present that could have masked potential beneficial effects of endothelial Arg1 ablation.

A limitation of our genetic approach to eliminate *Arg1* expression is that it may not be possible to selectively manipulate endothelial arginine concentrations in resistance arteries. In many vascular beds, resistance arteries exhibit heterocellular myoendothelial gap junctions (Dora et al. 2003; Sandow et al. 2012; Hilgers and De Mey 2009) through which arginine could diffuse from the smooth muscle to the endothelial cells and vice versa. Arginine diffusion through these pores may compensate for changes in endothelial arginine concentration elicited by, for example, decreased arginine degradation in endothelial cells. In endothelial cells, gap junctions are primarily formed of the connexin proteins CX37 (GJA4), CX40 (GJA5) and CX43 (GJA1) (Meens et al. 2015). Because the diabetic condition reduces their expression in vascular walls (Bobbie et al. 2010; Hou et al. 2008), reduced gap‐junction activity could explain limited repletion of arginine in this condition (Chennupati et al. 2014). This hypothesis implies that the myoendothelial gap junction‐dependent repletion of endothelial arginine is not sufficiently reduced to limit NOS activity irrespective of whether or not ARG1 is present in the endothelium. Unfortunately, this hypothesis is difficult to test, since inhibitors of gap‐junction activity are well known to be nonselective (Matchkov et al. 2004). The alternative genetic approach is also complex, since it would require the production of an endothelium‐specific triple knockout of C × 37, C × 40, and C × 43.

Both ARG1 and ARG2 are found in endothelium (Pernow and Jung 2013) and have been implicated in endothelial dysfunction. Toque et al. (Toque et al. 2013b) attributed a larger role to ARG1 than ARG2 in mediating hypertension. ARG1 also appears involved in diabetes as underscored in our study by the increased endothelial ARG1 staining in diabetic control mice and increased plasma arginine concentration in diabetic Arg1‐KO^Tie2^ mice.

One would assume that elimination of ARG1 should increase the circulating arginine concentration. However, in this study, such increase was only observed in diabetic Arg1‐KO^Tie2^ mice. The similar 30% increase in the summed concentration of amino acids suggests that the effect is not specific for arginine. In agreement, the arginine‐availability index (plasma [Arg]/([Orn]+[Lys])) did not change (Table 1). This index is a marker for the competitive strength of arginine over other basic amino acids for transport into endothelial cells (Morris et al. 2004). When plasma arginine was depleted acutely by ARG1 bolus injection, the arginine‐availability index dropped to ~25% of the original (Wijnands et al. 2015). In the present study, a similar intervention caused blood pressure to increase (Fig. 3A), implying that extracellular arginine availability had become limiting for blood pressure maintenance. However, even during this challenge, the increase in blood pressure was only ~30% of that achieved by inhibition of NOS by L‐NAME, implying an important role for intra‐endothelial arginine repletion or resynthesis by argininosuccinate synthase (ASS). This conclusion is underscored by our earlier findings in diabetic Ass‐KO^Tie2^ mice; the summed amino acid concentrations and the arginine‐availability index were similar to controls (Chennupati et al. 2014) as in this study. Whereas no endothelial dysfunction developed in control Ass‐KO^Tie2^ mice, it did in diabetic Ass‐KO^Tie2^ mice, showing that the contribution of resynthesis of arginine via ASS to NO synthesis became a limiting factor in diabetes (Chennupati et al. 2014). In the jejunum of untreated Arg1‐KO^Tie2^ mice, on the other hand, NOS3 was hypo‐phosphorylated on Thr495 and tended to be hyper‐phosphorylated on Ser1177 (Wijnands et al. 2014), suggesting that *Arg1* deficiency indeed increased NOS3 activity (Chen et al. 2008; Lin et al. 2003). In fact, we based our hypothesis that a limitation of the capacity of the endothelium to degrade arginine via ARG1 would improve arterial endothelium‐dependent relaxation. The combination of our earlier finding that the diabetic condition does limit the ASS‐mediated repletion of arginine and finding in the present study that endothelial *Arg1* elimination does not improve direct smooth muscle relaxation in (hyperglycemic) saphenous arteries implies that, although the endothelial arginine pool cannot be sufficiently repleted by surrounding smooth muscle cells, this pool is not sufficiently reduced to limit NOS activity in diabetic Arg1‐KO^Tie2^ mice.

EDH‐related arterial relaxing responses were reduced in diabetic mice, as shown earlier (Chennupati et al. 2014; Morikawa et al. 2005),. In mouse mesenteric arteries, endothelium‐derived H_2_O_2_ mediates these responses (Matoba et al. 2000). The main sources of H_2_O_2_ are superoxide dismutases (SODs) (Morikawa et al. 2003). The superoxide anion substrates of these SODs can be generated by a broad variety of enzymes such as the mitochondrial electron transfer chain, NADPH oxidases, xanthine oxidase, cyclooxygenases, lipoxygenase, and NO synthases (Fleming et al. 2001; Katusic 1996; Stroes et al. 1998). Superoxide anion production by NOS (EDH/H_2_O_2_ production by the endothelium) is markedly enhanced by “uncoupling” and de‐dimerization of the enzyme in response to shortage the co‐factor BH_4_ or the substrate (L‐Arg) (Munzel et al. 2005; Forstermann and Munzel 2006). Although we did not investigate the underlying mechanism for reduced EDH responses in mouse saphenous arteries during diabetes, substrate availability can be one of the factors. Accordingly, we expected that EDH‐dependent relaxation responses in Arg1‐KO^Tie2^ mice would be reduced. However, this was not observed. Other factors in superoxide/H_2_O_2_ generation may have contributed.

Diabetic mice displayed reduced smooth muscle sensitivity to exogenous NO. Despite comparable overall endothelium‐dependent relaxation, this was more pronounced in Arg1‐KO^Tie2^ than control mice. This may indicate increased EDNO production in Arg1‐KO^Tie2^ mice, which would be in line with our initial hypothesis that substrate abundance in endothelial cells increases NO production. Over time, this could have been compensated by a progressively reduced sensitivity of the arterial smooth muscle cells to NO.

In conclusion, deletion of *Arg1* using the Tie2 promoter did not affect MAP or heart rate in control mice. In addition, ex vivo studies of saphenous arteries from control and diabetic mice showed that arterial smooth muscle and endothelium‐dependent relaxing responses were largely unaffected by ablation of *Arg1* in the endothelium and hematopoietic cells. Comparison of the responses of endothelial *Ass* (our previous study) and *Arg1* ablation may suggest that only ASS*‐*generated arginine is accessible to NOS3 in this model (Flam et al. 2001).

### Data availability statement

All data generated or analyzed during this study are included in this published article (and its Supplementary Information files).

## Conflicts of interests

The authors declare that there are no conflicts of interest, financial or otherwise.

## Data Accessibility

## Supporting information




**Table S1**. Fasting blood glucose concentrations in male and female control and Arg1‐KO^Tie2^ mice before (basal value) and at the indicated times after streptozotocin treatment. All values are shown as means ± SEM.
**Table S2.** Effect of Arg1‐ablation on saphenous artery (SA) diameter and SA contractile responses in male mice. *E*_max_ values are expressed as % of the maximal response to noradrenaline (10 *µ*mol·L^*−*1^ NA).
**Table S3**. P‐values of comparisons of plasma amino acid concentrations between control and Arg1‐KO^Tie2^ mice, and normoglycemic and diabetic mice, respectively.
**Table S4.** *P*‐values of comparisons of relaxation responses as shown in Table 2.
**Table S5.** Effect of Arg1‐ablation on saphenous artery (SA) diameter, contractile, and relaxation responses in female mice.
**Figure S1**. Specificity of arginase‐1 antibody was demonstrated by simultaneous staining of liver tissue.
**Figure S2.** The effect of endothelial *Arg1* ablation on relaxation responses to ACh (0.01–10 *µ*mol·L^–1^) during K^+^‐ (40 mmol·L^–1^) induced contractions in saphenous arteries of 12‐ (panel A), 34‐ (panel B) week‐old normoglycemic and 22‐week‐old diabetic (panel C) male mice. Black squares: control mice; white circles: Arg1‐KO^Tie2^ mice.
