# Supplementary material for: Deletion of endothelial arginase 1 does not improve vasomotor function in diabetic mice
Source: Physiol Rep. 2018 Jun 10;6(11):e13717. doi: 10.14814/phy2.13717 (PMC5995309; doi:10.14814/phy2.13717)
Supplement: Supplementary file 1 — Table S1. Fasting blood glucose concentrations in male and female control and Arg1‐KOTie2 mice before (basal value) and at the indicated times after streptozotocin treatment. All values are shown as means ± SEM. Table S2. Effect of Arg1‐ablation on saphenous artery (SA) diameter and SA contractile responses in male mice. E max values are expressed as % of the maximal response to noradrenaline (10 µmol·L−1 NA). Table S3. P‐values of comparisons of plasma amino acid concentrations between control and Arg1‐KOTie2 mice, and normoglycemic and diabetic mice, respectively. Table S4. P‐values of comparisons of relaxation responses as shown in Table 2. Table S5. Effect of Arg1‐ablation on saphenous artery (SA) diameter, contractile, and relaxation responses in female mice. Figure S1. Specificity of arginase‐1 antibody was demonstrated by simultaneous staining of liver tissue. Figure S2. The effect of endothelial Arg1 ablation on relaxation responses to ACh (0.01–10 µmol·L–1) during K+‐ (40 mmol·L–1) induced contractions in saphenous arteries of 12‐ (panel A), 34‐ (panel B) week‐old normoglycemic and 22‐week‐old diabetic (panel C) male mice. Black squares: control mice; white circles: Arg1‐KOTie2 mice. [file PHY2-6-e13717-s001.docx]

**SUPPLEMENTAL DATA**

**Table S1**. **Fasting blood glucose concentrations** in male and female control and Arg1-KO^Tie2^ mice before (basal value) and at the indicated times after streptozotocin treatment. All values are shown as means ± SEM.

|  | **Basal value** | | **Time after STZ treatment** | | | | | |
| --- | --- | --- | --- | --- | --- | --- | --- | --- |
|  |  |  | **1 week** | | **4 weeks** | | **10 weeks** | |
|  | mmol.L^-1^ | n | mmol.L^-1^ | n | mmol.L^-1^ | n | mmol.L^-1^ | n |
| **Male mice** | | | | | | | | |
| Control | 8.1 ± 2.9 | 8 | 20.4 ± 0.9 | 11 | 24.2 ± 1.0 | 11 | 22.8 ± 0.8 | 10 |
| Arg1-KO^Tie2^ | 7.4 | 1 | 21.2 ± 2.4 | 6 | 25.2 ± 1.2 | 6 | 23.1 ± 0.6 | 5 |
| **Female mice** | | | | | | | | |
| Control | 8.9 ± 3.0 | 9 | 12.1 ± 1.0 | 8 | 13.8 ± 1.1 | 8 | 7.8 ± 1.0 | 8 |
| Arg1-KO^Tie2^ | 5.6 ± 0.3 | 2 | 11.5 ± 1.8 | 4 | 12.5 ± 1.0 | 4 | 7.6 ± 0.9 | 4 |

**Table S2. Effect of Arg1-ablation on saphenous artery (SA) diameter and SA contractile responses** in male mice**.** E_max_ values are expressed as % of the maximal response to noradrenaline (10 µmol.L^-1^ NA). All values are shown as mean ± SEM. n.d = not determined. *: P<0.05 compared to arteries of 12- and 34-week old mice under the same condition (unpaired t-test).

|  | **Control** | | | **Arg1-KO^Tie2^** | | |
| --- | --- | --- | --- | --- | --- | --- |
| **Optimal diameter (µm)** |  | | |  | | |
| 12-week-old mice | 251 ± 3 | | | 237 ± 11 | | |
| 34-week-old mice | 247 ± 4 | | | 241 ±  3 | | |
| 22-week-old diabetic mice | 224 ± 4 | | | 253 ± 12 | | |
| **Contraction to 10 µM NA (N/m)** |  | | |  | | |
| 12-week-old mice | 2.9 ± 0.1 | | | 2.9 ± 0.2 | | |
| 34-week-old mice | 2.9 ± 0.3 | | | 3.1 ± 0.3 | | |
| 22-week-old diabetic mice | 3.5 ± 0.3 | | | 3.6 ± 0.3 | | |
|  | **pEC_50_** | **E_max_ (%)** | **n** | **pEC_50_** | **E_max_ (%)** | **n** |
| **Contractions to 40 mM K^+^** |  |  |  |  |  |  |
| 12-week-old mice | n.d. | 109 ± 5 | 7 | n.d. | 104 ±  4 | 4 |
| 34-week-old mice | n.d. | 100 ± 5 | 6 | n.d. | 104 ± 11 | 6 |
| 22-week-old diabetic mice | n.d. | 104 ± 2 | 7 | n.d. | 111 ±  6 | 4 |
| **PHE-induced contraction** | | | | | | |
| ***12-week-old mice*** |  |  |  |  |  |  |
| Without inhibitors | 5.7 ± 0.1 | 94 ± 4 | 5 | 5.8 ± 0.1 | 100 ± 4 | 3 |
| INDO | 5.6 ± 0.1 | 90 ± 6 | 6 | 5.7 ± 0.1 | 101 ± 5 | 5 |
| INDO + L-NAME | 5.7 ± 0.1 | 101 ± 3 | 6 | 5.7 ± 0.1 | 106 ± 5 | 5 |
| ***34-week-old mice*** | | | | | | |
| Without inhibitors | 5.7 ± 0.1 | 102 ± 8 | 5 | 5.9 ± 0.2 | 89 ± 6 | 4 |
| INDO | 5.6 ± 0.1 | 94 ± 10 | 5 | 5.6 ± 0.1 | 85 ± 8 | 4 |
| INDO + L-NAME | 5.7 ± 0.1 | 97 ±  8 | 5 | 5.9 ± 0.1 | 98 ± 8 | 4 |
| ***22-week-old diabetic mice*** | | | | | | |
| Without inhibitors | 5.8 ± 0.1 | 104 ± 2 | 8 | 5.7 ± 0.1 | 95 ± 3 | 5 |
| INDO | 5.6 ± 0.1 | 104 ± 3 | 8 | 5.6 ± 0.1 | 95 ± 3 | 5 |
| INDO + L-NAME | 5.6 ± 0.1 | 99 ± 3 | 8 | 5.8 ± 0.2 | 101 ± 4 | 5 |

**Table S3**. **P-values of comparisons of plasma amino acid concentrations between control and Arg1-KO^Tie2^ mice, and normoglycemic and diabetic mice, respectively.** Means of amino acid concentrations are given in Table 1. Significant differences (P < 0.05) are given in bold; trends (0.05 < P ≤ 0.075) are shown in italics. AAI = arginine availability index**.**

| **Plasma amino acids** | **Control vs**  **Arg1-KO^Tie2^** | **STZ control vs**  **STZ-Arg1-KO^Tie2^** | **Control vs**  **STZ-control** | **Arg1-KO^Tie2^ vs**  **STZ-Arg1-KO^Tie2^** |
| --- | --- | --- | --- | --- |
| Alanine | 0.330 | 0.414 | 0.890 | 0.105 |
| Arginine | 0.512 | **0.030** | 0.396 | 0.354 |
| Asparagine | 0.504 | *0.067* | 0.587 | 0.167 |
| Ornithine | 0.286 | 0.355 | 0.659 | 0.098 |
| Citrulline | 0.386 | 0.443 | **0.036** | **0.003** |
| Glutamic Acid | 0.695 | 0.476 | 0.694 | 0.194 |
| Glutamine | 1.000 | 0.490 | 0.795 | 0.593 |
| Glycine | 0.728 | 0.151 | 0.196 | 0.734 |
| Histidine | 0.226 | 0.717 | 0.203 | 0.762 |
| Isoleucine | 0.465 | 0.607 | 0.139 | 0.082 |
| Leucine | 0.802 | 0.635 | 0.154 | 0.101 |
| Lysine | 1.000 | 0.211 | 0.599 | *0.053* |
| Methionine | 0.560 | 0.925 | 0.509 | 0.286 |
| Phenylalanine | 0.179 | *0.058* | *0.057* | 0.213 |
| Taurine | 0.955 | **0.001** | 0.577 | **0.025** |
| Serine | 0.504 | 0.367 | 0.907 | 0.282 |
| Threonine | 0.513 | 0.245 | 0.791 | 0.103 |
| Tryptophan | 0.929 | 0.752 | *0.054* | **0.019** |
| Tyrosine | 0.263 | 0.923 | 0.817 | *0.075* |
| Valine | 0.107 | 0.641 | 0.258 | 0.097 |
| ΣAA | 0.816 | *0.055* | 0.517 | 0.103 |
| AAI | 0.7835 | 0.226 | 0.791 | 0.178 |

**Table S4. P-values of comparisons of relaxation responses as shown in Table 2.** Significant differences (P-values < 0.05) are given in bold. n.d.: not determined.

|  | **Control vs Arg1-KO^Tie2^** | |
| --- | --- | --- |
|  | **pEC_50_** | **E_max_%** |
| **12-week-old mice** | | |
| Without inhibitors | 0.213 | 0.409 |
| INDO | 0.501 | 0.146 |
| INDO + L-NAME | 0.351 | 0.940 |
| Relaxation to SNP | 0.499 | 1.000 |
| Relaxation to EDNO | 0.213 | 0.577 |
| **34-week–old mice** | | |
| Without inhibitors | 0.518 | 0.749 |
| INDO | 0.718 | 0.872 |
| INDO + L-NAME | n.d. | 0.385 |
| Relaxation to SNP | 0.740 | 0.508 |
| Relaxation to EDNO | 0.330 | 0.919 |
| **22-week-old diabetic mice** | | |
| Without inhibitors | 0.635 | 0.691 |
| INDO | 1.000 | 0.515 |
| INDO + L-NAME | n.d. | 0.900 |
| Relaxation to SNP | **0.045** | 0.200 |
| Relaxation to EDNO | 0.369 | 0.369 |

**Table S5. Effect of Arg1-ablation on saphenous artery (SA) diameter, contractile and relaxation responses in female mice.**

**Maximal contraction (**E_max_) values are expressed as % of the maximal response to noradrenaline (10 µmol.L^-1^ NA). Relaxation responses to ACh (0.01-10 µmol.L^-1^) were determined in PHE (10 µmol.L^-1^) or K^+^ (40 mmol.L^-1^) pre-contracted arteries in the presence or absence of the prostacyclin inhibitor INDO (10 µmol.L^-1^) and the NOS3 inhibitor L-NAME (100 µmol.L^-1^). E_max_ is expressed as % reduction of the maximal contractile response to 10 µmol.L^-1^ PHE except for EDNO responses (% reduction of maximal contractile response to 40 mmol.L^-1^ K^+^). Maximal relaxation to an NO-donor (SNP, 0.01-10 µmol.L^-1^) is determined in the presence of L-NAME (100 µmol.L^-1^) and INDO (10 µmol.L^-1^). All values are shown as means ± SEM.

|  | **Control** | | | | **Arg1-KO^Tie2^** | | |
| --- | --- | --- | --- | --- | --- | --- | --- |
| **Optimal diameter (µm)** |  | | | |  | | |
| 12-week-old mice | 235 ± 12 | | | | 247 ± 11 | | |
| 34-week-old mice | 252 ± 14 | | | | 232 ± 6 | | |
| **Contraction to 10 µM NA (N/m)** |  | | | |  | | |
| 12-week-old mice | 2.7 ± 0.2 | | | | 2.6 ± 0.2 | | |
| 34-week-old mice | 2.9 ± 0.2 | | | | 2.8 ± 0.3 | | |
| **PHE-induced contraction** | | | | | | | |
| ***12-week-old mice*** | pEC_50_ | E_max_% | n | | pEC_50_ | E_max_% | n |
| Without inhibitors | 5.9 ± 0.2 | 96 ± 6 | 8 | | 5.8 ± 0.2 | 106 ± 8 | 6 |
| INDO | 5.6 ± 0.1 | 90 ± 6 | 8 | | 5.7 ± 0.1 | 101 ± 5 | 6 |
| INDO + L-NAME | 5.7 ± 0.1 | 101 ± 3 | 8 | | 5.7 ± 0.1 | 106 ± 5 | 6 |
| ***34-week-old mice*** | | | | | | | |
| Without inhibitors | 5.8 ± 0.2 | 88 ± 10 | | 7 | 5.9 ± 0.2 | 101±11 | 6 |
| INDO | 5.7 ± 0.1 | 90 ± 12 | | 7 | 5.8 ± 0.1 | 90 ± 12 | 6 |
| INDO + L-NAME | 5.7 ± 0.1 | 92 ±  9 | | 7 | 5.9 ± 0.1 | 92 ± 9 | 6 |
| **ACH-induced relaxation** | | | | | | | |
| **12-week-old mice** | pEC_50_ | E_max_% | | n | pEC_50_ | E_max_% | n |
| Without inhibitors | 6.7 ± 0.1 | 97 ± 2 | | 7 | 6.7 ± 0.1 | 90 ± 2 | 6 |
| INDO | 6.9 ± 0.1 | 93 ± 2 | | 8 | 6.7 ± 0.1 | 90 ± 1 | 6 |
| INDO + L-NAME | 6.6 ± 0.4 | 65 ± 8 | | 7 | 6.4 ± 0.2 | 51 ± 5 | 6 |
| **Relaxation to SNP** |  |  | |  |  |  |  |
| INDO + L-NAME | 7.3 ± 0.1 | 98 ± 8 | | 5 | 7.4 ± 0.1 | 97 ± 3 | 5 |
| **34-week–old mice** | | | | | | | |
| Without inhibitors | 6.9 ± 0.1 | 87 ± 3 | | 7 | 6.9 ± 0.1 | 93 ± 3 | 5 |
| INDO | 6.9 ± 0.1 | 87 ± 2 | | 7 | 7.0 ± 0.1 | 91 ± 3 | 5 |
| INDO + L-NAME | n.d | 34 ± 4 | | 7 | n.d | 37 ± 4 | 5 |
| **Relaxation to SNP** | | | | | | | |
| INDO + L-NAME | 7.4 ± 0.1 | 96 ± 1 | | 4 | 7.5 ± 0.1 | 96 ± 1 | 6 |

**Supplemental Figures**


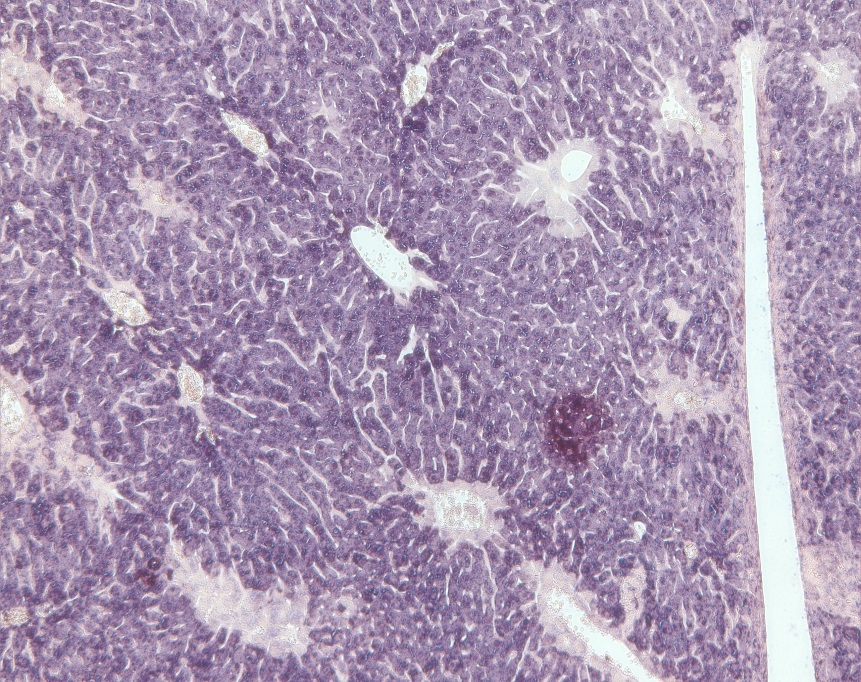

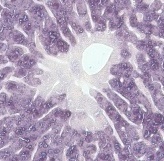


*

CV

***Figure S1****.* Specificity of arginase-1 antibody was demonstrated by simultaneous staining of liver tissue. Magnification: arginase 1 is expressed in all hepatocytes except for one or two cell layers (marked by an asterisk) around the central veins (CV).

***Figure S2****.* The effect of endothelial *Arg1* ablation on relaxation responses to ACh (0.01-10 µmol.L^-1^) during K^+^- (40 mmol.L^-1^) induced contractions in saphenous arteries of 12- (panel A), 34- (panel B) week-old normoglycemic and 22-week-old diabetic (panel C) male mice. Black squares: control mice; white circles: Arg1-KO^Tie2^ mice. All arteries were treated with indomethacin (INDO, 10 µmol.L^-1^). Values are shown as means ± SEM (n=4-8; for the number of animals per individual experiment, see Table S2).
